# A Community-Based Study on Functional Disability and Its Associated Factors Among Elderly Individuals in a Rural Setting in Northeastern India

**DOI:** 10.7759/cureus.13309

**Published:** 2021-02-12

**Authors:** Gajendra K Medhi, Vizovonuo Visi, Parash J Bora, Jogesh Sarma, Prasanta Borah, Jagdish Mahanta, Himashree Bhattacharyya, Star Pala

**Affiliations:** 1 Community Medicine, North Eastern Indira Gandhi Regional Institute of Health and Medical Sciences, Shillong, IND; 2 Pulmonary Medicine, Gauhati Medical College, Guwahati, IND; 3 Epidemiology and Nutrition, Indian Council of Medical Research (ICMR) - Regional Medical Research Centre, North-Eastern (NE) Region, Dibrugarh, IND; 4 Internal Medicine: Infectious Disease, Indian Council of Medical Research (ICMR) - Regional Medical Research Centre, North-Eastern (NE) Region, Dibrugarh, IND

**Keywords:** activity of daily living (adl), functional disability, elderly

## Abstract

Background

Functional disability in older adults is common and adversely impacts the quality of life. Given the paucity of population-based data, the present analysis attempted to determine the prevalence and factors associated with functional disability in basic activities of daily living (ADLs) among the elderly population in a rural setting of Northeastern state of India

Methods

A total of 430 elderly were recruited in a population-based cross-sectional study among elderly individuals (≥60 years) during the period 2013-2016 in rural areas of the Dibrugarh district of Northeastern India. The Barthel index was used to measure ADL. Anyone with a Barthel index score <100 (or having limitations in one or more ADL items) were considered as having a functional disability. The analysis of variance (ANOVA) test and the binary logistic regression analysis were used to examine the factors associated with functional disability.

Results

Overall, 43.7% % (Male 42.9%, Female 44.5%) of the participants had a functional disability. Increasing age, being widowed, having no formal education, being underweight (body mass index (BMI)<18.5 kg/m^2^), and increasing numbers of morbidities were significantly associated with functional disability among the elderly in this study in age and gender-adjusted logistic regression analysis. Being ≥80 years was associated with a three-fold greater risk (OR=31.72, 95% CI=1.3-6.91) of functional disability than the youngest age group (60-69 years). On the other hand, the presence of more than five morbidities was associated with a nearly 20 times increased risk (OR=19.61, 95% CI=9.01-42.68) than those with zero to two morbidities.

Conclusion

A high proportion of the rural elderly residents of Dibrugarh had a functional disability. The study provides epidemiological evidence of the risk factors of functional disability in this setting. This epidemiological information may be useful for developing prevention strategies to reduce the burden of functional disability.

## Introduction

Population aging is a global phenomenon, and India is also no exception to this phenomenon. India is experiencing constant growth in the size and proportion of older persons in its population [1]. The proportion of elderly persons (60+) in the population of India rose from 5.61% in 1961 to 8.6% in 2011, which is further projected to gradually rise to approximately 20% in 2050 [1-2]. Ensuring health and wellbeing to this ever-increasing section of the population is an enormous public health challenge because it is well-known that older people are more susceptible to multiple comorbidities and physical and cognitive decline, thereby increasing the need for greater health services and long-term care [3-4]. Thus, it is desirable that people maintain good health and high functioning into old age, both for the interest of older people themselves and for society as a whole [4].

Physical function is recognized as an important indicator of health and quality of life in older people [2,5]. Maintenance of good functional status is considered a crucial component of healthy aging [5]. Activities of daily living (ADLs) is an index commonly used to measure physical functioning [4,6]. Basic ADLs encompass common everyday tasks (such as eating, bathing, dressing, mobility, etc.) that are required for maintaining an independent life, performing normal daily activities for basic needs, or maintaining health and well-being [2,7-8]. Functional assessment is an integral part of the multidisciplinary evaluation of the elderly [9]. A comprehensive assessment of functional status is a fundamental aspect for providing care to older people [8]. A community-based assessment of ADL functioning among the elderly can help understand the burden of functional disability and predicts the need for assistance for this age group [8]. Further, it is also important to understand the factors associated with functional disability in order to design and implement interventions to improve their functional health [8].

Although there are some community-based studies available on functional status among older people in India, there is a paucity of such community-based data from the northeastern region of India, especially for the rural population where more than 70% of its elderly people reside [1-2]. With this background, this study aimed to assess the prevalence and factors associated with functional disability in ADL among elderly people (60 years and above) in a rural area of Assam, a northeastern state of India.

## Materials and methods

The study was a cross-sectional community-based study conducted in Dibrugarh district of Assam, India, during the period 2013-2016 among elderly individuals aged ≥60 years. The primary objective of the study was to evaluate the quality of life among elderly people. As part of the study, data on the functional status of elderly individuals were also obtained. We used this data set to assess the prevalence and factors associated with functional disability among the elderly subjects. The sampling method has been already described elsewhere [10]. Briefly, participants of the study were recruited into the study using a multi-stage sampling design. In the first stage, two development blocks out of seven were selected randomly from the total list of development blocks in the district. In the last stage, seven villages from each selected block were selected randomly for conducting the study. All community-dwelling individuals 60 years and above were eligible to participate in the study. Data were collected through house-to-house visits. Eligible individuals who were available in the households during our study team visit were recruited into the study. A total number of 430 eligible individuals were recruited in the study.

Ethical approval to conduct the study was obtained from the Institutional Ethical Committee of Regional Medical Research Centre (RMRC), Dibrugarh. Written informed consent was taken from all the respondents before data collection.

Face-to-face interviews were conducted using a predesigned and pretested questionnaire to collect data on sociodemographic variables and chronic morbidities. Height and weight were measured as per the guidelines of the World Health Organization (WHO) [11]. The weight and height were recorded to a minimum of 0.5 kg and 0.5 cm, respectively. The body mass index (BMI) of participants was calculated using the formula: weight (kg)/height (m^2^). Participants were divided into three groups for both men and women (underweight: BMI<18.5 kg/m^2^, normal weight: BMI 18.5-24.99 kg/m^2^, and overweight: BMI ≥25 kg/m^2 ^[11]. Morbidities were determined based on participants’ self-reported diagnosis or from self-reported symptoms-based measures as per the protocol of the study.

Activities of daily living (ADL)

The ADLs of elderly individuals was measured using the Barthel index scale [12]. This index is a widely used simple rating scale to measure the levels of functional capacity of individuals necessary for independent living in 10 basic areas of ADLs (12, 13, 14, 15). We used the Barthel index to measure participants’ self-reported ability to perform the following 10 ADL items, viz., feeding, bathing, grooming, dressing, bowel control, bladder control, toilet use, transferring (bed to chair and back), mobility on a level surface, and mobility on stairs. The total score in all the 10 items ranges from 0 to 100, with a higher score indicating greater independence [2]. A score of 0 indicates complete dependency in all 10 ADLs, whereas a score of 100 indicates complete independence. In this study, anyone with a Barthel index score <100 (or having limitations in one or more ADL items) was considered as having functional disability [13]. Participants were categorized into four groups based on the relative severity of disability as indicated by Barthel index scores: i) High disability (Barthel score: 0-49), ii) Moderate disability (Barthel score: 50-89, iii) Mild disability (Barthel score: 90-99).

Statistical analysis

Data were entered and analyzed using the Statistical Package for the Social Sciences (SPSS) Version 21 (IBM Corp. Armonk, NY). One-way analysis of variance (ANOVA) and age/gender-adjusted binary logistic regression analysis were used to examine the associations between dependent variables (functional disability) and other independent variables such as age, gender, marital status, BMI, education, income, and the number of morbidities. For logistic regression analysis, individuals were dichotomized into two groups according to ADL status. Anyone who scored <100 on the Barthel index scale was considered as having a functional disability. A P-value equal to or less than 0.05 was considered statistically significant for all the statistical procedures.

## Results

A total of 430 elderly individuals participated in this study, out of which 210 (48.8%) were male and 220 (51.2%) were females. The mean age of the participants was 68.71±7.42 years (Table 1).

**Table 1:** Characteristics of participants NB: BMI data is available from 397 subjects, as data on weight and height is not available for 33 subjects. BMI: body mass index

Variables (N=430)		n (%)
Age groups (in years)	60-69	250 (58.1)
	70-79	138 (32.1)
	80+	42 (9.8)
	Men Age±SD	68.71±7.42
Gender	Male	210 (48.8)
	Female	220 (51.2)
Educational status	No formal education	131 (30.5)
	Upto high school	224 (52.1)
	Beyond high school	75 (17.4)
Marital status	Married	252 (58.6)
	Widowed	173 (40.2)
	Separated/divorced	2 (0.5)
	Unmarried	3 (0.7)
BMI (kg/m^2^) (N=397)	Underweight	127 (32)
	Normal weight	214 (53.9)
	Overweight	56 (14.1)
Numbers of morbidities	0-2	93 (21.6)
	3-5	205 (47.7)
	>5	132 (30.7)
Total		430

Overall, 188 (43.7%) of the participants had a functional disability in one or more ADL items. In total, 56.3% of participants had no ADL disability in any ADL items. A total of 139 (32.2%) participants obtained a Barthel score of 90-99, whereas only seven (1.6%) obtained a score of less than 50. No statistically significant gender differences were observed in the prevalence of functional disability and mean Barthel score (Table 2). 

**Table 2:** Prevalence of functional disability and mean Barthel score

	Male n (%)	Female n (%)	Total n (%)
Prevalence of functional disability			
No functional disability	120 (57.1)	122 (55.5)	242 (56.3)
Functional disability	90 (42.9)	98 (44.5)	188 (43.7)
P-value	0.124		
Mean ADL score			
0-49	3 (1.4)	4 (1.8)	7 (1.6)
50-89	21 (10)	21 (9.5)	42 (9.8)
90-99	66 (31.4)	73 (33.2)	139 (32.3)
100	120 (57.2)	122 (55.5)	242 (56.3)
Total	210 (48.8)	220 (51.2)	430 (100)
P-value	0.157		

Table 3 shows the mean Barthel scores and prevalence of functional disability in the area of ADLs according to age, gender, education, income, marital status, BMI, and the number of morbidities. The prevalence of functional disability was found to increase with increasing age, and the highest prevalence was observed in ≥80 years of age (73.8%). We see a significant inverse relationship between mean Barthel scores and age, the mean score being lowest among ≥80 years of age. No statistically significant gender difference was observed in the functional disability. Educational status was significantly associated with functional disability. Those who had no formal education had a significantly higher prevalence of functional disability as compared with other educational groups. Compared with currently married individuals, the widowed had poorer ADL status, both in terms of the prevalence of functional disability and mean Barthel scores. BMI showed a U-shaped relationship with functional disability. Those who were underweight (BMI<18.5 kg/m^2^) or overweight/obese (BMI≥25 kg/m^2^) had a significantly poorer ADL status than individuals with normal weight. The number of morbidities was found to be significantly associated with functional disability. The prevalence of functional disability increased with increasing counts of morbidities. On the other hand, the mean Barthel score decreased with increasing counts of morbidities.

**Table 3:** Mean Barthel score and prevalence of functional disability according to age, gender, education, marital status, BMI, and number of morbidities BMI: body mass index

Variables	Mean Barthel score ± SD	Prevalence of functional disability n (%)
Age groups (in years)		
60-69	96.06±9.8	85 (34)
70-79	93.11±12.67	72 (52.2)
80 years and above	86.90±16.89	31 (73.8)
All Age	94.22±11.95	188 (43.7)
P-value	.000	.000
Gender		
Male	94.09±11.80	90 (42.9)
Female	94.34±12.12	98 (44.5)
P-value	0.832	0.724
Education		
No formal education	93.20±12.31	68 (51.9)
Upto high school	94.26±11.95	96 (42.9)
Beyond high school	95.86±11.28	24 (32.0)
P-value	0.307	0.020
Marital Status		
Married	95.35±10.89	91 (36.1)
Widowed	92.60±13.25	95 (54.9)
P-value	0.020	.000
BMI (N=397)		
Underweight	94.60±8.47	62 (49.2)
Normal weight	97.15±4.81	72 (33.8)
Overweight and obese	95.62±6.94	22 (39.3)
P-Value	0.019	0.020
Number of morbidities		
0-2	98.87±3.90	10 (10.8)
3-5	95.60±9.54	81 (39.5)
>5	88.78±16.34	97 (73.5)
P-value	0.770	0.000

Table 4 shows that a high prevalence (Barthel score: 0-49) and moderate level (Barthel score: 50-89) of disability increased with increasing age, the prevalence being the lowest in the 60-69 years' age group and highest in the ≥80 years age group. On the other hand, the prevalence of mild disability (Barthel score 90-99) was similar in the age group of 70-79 and ≥80 years.

**Table 4:** Distribution of Barthel scores according to age

Age groups	Barthel score			
	0-49	50-89	90-99	100
60-69 (n=250)	2 (0.2)	16 (6.4)	67 (26.8)	165 (66)
70-79 (n=138)	3 (2.2)	14 (10.1)	55 (39.9)	66 (47.8)
80+ (n=42)	2 (4.8)	12 (28.6)	17 (40.5)	11 (26.2)
All age (N=430)	7 (1.6)	42 (9.8)	139 (32.3)	242 (56.3)
P-value	P=0.000			

In the age and gender-adjusted logistic regression analysis, functional disability was significantly associated with factors such as increasing age, having no formal education, being widowed, being underweight (BMI<18.5 kg/m^2^), and increasing numbers of morbidities (Table 5).

**Table 5:** Results of logistic regression analysis showing factors associated with functional disability * Significant p-value (0.05), **Significant p-value (P<0.01) COR: crude odds ratio, AOR: adjusted odds ratio (adjusted for age and gender), BMI: body mass index

Variables	COR (95%CI)	AOR (95% CI)
Age groups (in years)		
60-69	1 (Reference)	1(Reference)
70-79	2.58 (1.20-5.55)**	1.53 (.94-2.50)
80 years and above	5.47 (2.62-11.42)**	3.00 (1.30-6.91)**
Sex		
Male	1(Reference)	1(Reference)
Female	1.07 (0.73-1.56)	1.08 (0.65-1.79)
Education		
No formal education	2.2 (1.26-4.15)**	1.86 (0.99-3.48)*
Upto high school	1.59 (0.917-2.77)	1.42 (0.80-2.51)
Beyond high school	1(Reference)	1(Reference)
Marital status		
Married	1(Reference)	1(Reference)
Widowed	2.15 (1.45-3.19)**	1.87 (1.20-2.93)**
BMI		
Underweight	1.89 (1.21-2.97)**	1.64 (1.03-2.62)*
Normal weight	1(Reference)	1(Reference)
Overweight and obese	1.26 (0.69-2.32)	1.31 (0.07-2.46)
Number of morbidities		
0-2	1 (Reference)	1(Reference)
3-5	5.42 (2.65-11.06)**	4.74 (2.29-9.797)**
>5	23.00 (10.74-49.25)**	19.61 (9.01-42.68)**

## Discussion

This population-based study aimed to determine the prevalence and factors associated with ADL-based functional disability among elderly individuals in a rural setting in India. To the best of our knowledge, this is the first population-based study to evaluate ADL status among rural elderly individuals in the northeastern region of India. Overall, the prevalence of functional disability was 43.7% in this study. Medhi et al. (2020) reported a lower prevalence (34.7%) of functional disability among urban elderly (60+) residents in a recent community-based study, which also used the same instrument to assess ADL [2]. The better functional health among elderly people residing in an urban area in comparison to that of rural elderly could be because urban residents have better access to health care, have better logistic support in the form of transportation, have better financial support in the form of retirement benefits, or maybe because of their less dependence on the physical effort needed to complete certain tasks [13].

Other community-based studies conducted in other parts of the county using similar study instruments have reported the prevalence of ADL-based functional disability ranging from 16.2% to 53.6% [8,13-17]. Such wide variations in the prevalence of functional disability observed among participants between different settings could be because of differences in the age structure of participants, socioeconomic differences, and differences in health care and the social support system [2,8,13-17]. Such variations could also be explained by the fact that studies were conducted in different time periods.

According to the results of this analysis, an increase in age, being widowed, of lower education status, BMI other than normal (BMI: 18.50-24.99), and an increase in the number of morbidities were found to be associated with functional disability. As reported in previous studies in India, increasing age was found to be an important factor associated with functional disability in this study [8,13-17]. In this study, individuals in the oldest age group of ≥80 years were three times more likely to be functionally disabled than individuals in the youngest age group of 60-69 years. Severe to moderate functional disability also showed a similar correlation with chronological aging, with the functional disability rate being the highest in the ≥80 years age group. Overall, our results suggest that there may be a further escalation of disability burden in the community as the numbers and proportion of the oldest old persons (≥80 years) is also gradually increasing in the population due to increased life expectancy at an older age [1]. One notable finding of the study is that nearly 26% among ≥80 years of age were disability-free and many of them also had only a mild disability, which suggests that older people can remain functional fit even into very old age.

While previous studies reported a higher prevalence of functional disability among elderly females than elderly males in India [13,15,17], we could not detect any such gender disparity in this study. The finding of the study, which is consistent with previous reports from this region, suggests that elderly people of both genders in this region are equally vulnerable to functional disability [2,17-18]. In our study, being widowed as opposed to married was associated with greater odds of functional disability after adjustment for age and gender, which is consistent with previous reports [2,15,19-20]. Mutual support and help between married couples probably influence the positive health outcomes among married couples during older age [19-20].

Consistent with other previous reports, we also observed an inverse educational gradient in the prevalence of functional disability [2,18-20]. The lowest educated group had the highest and the highest educated group had the lowest prevalence of functional disability. Differences in the level of health awareness and health behaviors among individuals with different levels of educations might explain such educational gradients [19-20]. Furthermore, the opportunity to get a job with better income is likely to be less among illiterates or less educated people, which may adversely influence their health spending to keep them healthy [19-20].

In this study, a U-shaped relationship between BMI and functional disability was observed. Both underweight and overweight individuals had a higher prevalence of disability than normal-weight individuals. Such a finding was also reported by Medhi et al. in a previous study among the urban elderly in this region [18]. However, in age and gender-adjusted analysis, only the underweight showed a significantly increased risk of disability, which is consistent with previous studies [21]. Older adults who are underweight have a higher risk of sarcopenia and frailty, which may consequently lead to greater functional disability [17,21-22]. Our result suggests that we should pay more attention to the underweight, rather than the overweight, for the prevention of disability among the elderly [21-22]. However, our results on the relationship between functional disability and overweight/obesity should be interpreted cautiously because our results are not in accordance with other previous studies suggesting overweight/obesity as a risk factor of poor physical functioning [21].

Similar to previous studies, the risk of disability was found to progressively increase with the increasing number of morbidities in this study [2,23-25]. It is increasingly evident that multimorbidities have a greater impact on functional disability than individual morbidities, indicating an additive or synergistic effect of various combinations of morbidities on disability [26-28]. The presence of multiple chronic diseases may have various complex interactions causing greater difficulty for elderly individuals in performing daily activities requiring assistance from others [26]. The increased disability risk observed with the cumulative effect of various morbidities highlights the importance of taking into account multimorbidity when investigating the disability burden among the elderly [27].

Limitations

The limitations of the study should be considered in interpreting the results of this study. The inherent limitations of a cross-sectional study should be kept in mind in interpreting the causal relationships. The findings of the study are not generalizable to the entire rural population of the country, as this study was conducted only in one district. As data on ADL were self-reported, there could be a possibility of bias. However, studies have shown that self-reported disability is congruent with disabilities diagnosed by medical service [29]. We also relied mostly on self-reporting for ascertaining morbidities, which may lead to some reporting bias because of the high prevalence of the under-diagnosis of diseases in the rural population.

## Conclusions

The first-ever population-based study among the rural elderly population in the Northeastern region of India shows that more than 40% of elderly people had some form of functional disability in terms of difficulties in performing basic activities of daily living. Given the current trend of population aging in India, the burden of functional disability is expected to further increase. Functional disability was significantly associated with factors such as increasing age, widowhood, having no formal education, underweight, and an increase in the number of morbidities. Proactive measures need to be initiated to ensure proper care and support to increasing numbers of functionally disabled individuals, especially targeting vulnerable groups identified in this study such as the oldest-old and less educated elderly people. Public health programs should strengthen their efforts for the prevention of disability through primary prevention efforts to compress morbidity to extreme old age, through the adequate treatment and management of morbidities, especially multimorbidities, and by giving attention to the adequate nutrition of elderly people. Further studies with more methodological rigor should be conducted for a deeper insight into the relations between the factors identified in this study and disability for developing more effective public health actions.
